# A comparative blind study between skin biopsy and seed amplification assay to disclose pathological α-synuclein in RBD

**DOI:** 10.1038/s41531-023-00473-5

**Published:** 2023-03-04

**Authors:** R. Liguori, V. Donadio, Z. Wang, A. Incensi, G. Rizzo, E. Antelmi, F. Biscarini, F. Pizza, Wq Zou, G. Plazzi

**Affiliations:** 1 IRCCS Istituto delle Scienze Neurologiche di Bologna, UOC Clinica Neurologica, Bologna, Italy; 2 Dipartimento di Scienze Biomediche e Neuromotorie, Università di Bologna, Bologna, Italy; 3 Departments of Pathology and Neurology, Case Western Reserve University School of Medicine, Cleveland, OH 44106 USA; 4 Dipartimento di Neuroscienze, Biomedicina e Movimento, Università di Verona, Verona, Italy; 5 Department of Biomedical, Metabolic and Neural Sciences, University of Modena and Reggio-Emilia, Modena, Italy

**Keywords:** Diagnostic markers, Parkinson's disease

## Abstract

To compare the diagnostic accuracy of the immunofluorescence (IF) technique and aSyn-seed amplification assay (aSyn-SAA) of skin and cerebrospinal fluid (CSF) in disclosing pathological α-syn in idiopathic idiopathic REM sleep behavior disorder (iRBD) as early phase of a synucleinopathy. We prospectively recruited 41 patients with iRBD and 40 matched clinical controls including RBD associated with type 1 Narcolepsy (RBD-NT1, 21 patients), iatrogenic causes (2 pt) or OSAS (6 pt) and 11 patients with peripheral neuropathies. IF from samples taken by skin biopsy and aSyn-SAA from skin and CSF samples were analysed blinded to the clinical diagnosis. IF showed a good diagnostic accuracy (89%) that was lower in the case of skin and CSF-based aSyn-SAA (70% and 69%, respectively) because of a lower sensitivity and specificity. However, IF showed a significant agreement with CSF aSyn-SAA. In conclusion, our data may favor the use of skin biopsy and aSyn-SAA as diagnostic tools for a synucleinopathy in iRBD.

## Introduction

Idiopathic REM sleep behavior disorder (iRBD) has the highest positive predictive value for synucleinopathies^1,2^ but RBD can be also secondary to other disorders such as type 1 Narcolepsy (RBD-NT1), a central hypersomnia characterized by a loss of hypocretin neurons but not related to a neurodegenerative process^2^. Immunofluorescence (IF) technique^3,4^ and real-time quaking-induced conversion (RT-QuIC) on cerebrospinal fluid (CSF)^5^ and, more recently, skin samples^6,7^ are described diagnostic tools able to identify in vivo pathological α-synuclein (α-syn) although they explore different aspects of misfolded α-syn. IF allows to detect the morphology of pathological α-syn aggregates in skin nerves by antibodies directed against the phosphorylated α-syn (p-syn), whereas RT-QuIC or aSyn-seed amplification assay (aSyn-SAA) can determine their seeding activity, a prion-like biological trait of misfolded proteins. In the manuscript, we will use the term aSyn-SAA which is the established unified name to refer to assays previously known as RT-QuIC or PMCA. Thus, a direct comparison of these two techniques is important as their diagnostic accuracy may differ in iRBD as already described in full-blown variants of synucleinopathy^7^.

The aim of this study is to compare the diagnostic accuracy of skin IF and skin and CSF-based aSyn-SAA in disclosing pathological α-syn in iRBD as early phase of a synucleinopathy.

## Results

iRBD patients and controls were matched for sex-age and RBD duration although patients with iatrogenic and NT1-RBD presented RBD onset in a younger age (Table 1). As expected, nigro-striatal DatScan and neuropsychological abnormalities were more prevalent in patients with iRBD although these tests were performed in only few controls (Table 1). Abnormal nigro-striatal DatScan was found in 3 RBD-NT1 patients: two of them showed negative aSyn-SAA while the other patient with a young-onset parkinsonism presented a positive aSyn-SAA. Interestingly, almost half of iRBD patients reported hyposmia which was absent in the controls. Hyposmia was highly prevalent in iRBD patients with positive IF in skin nerves (98%) and positive aSyn-SAA both considering skin and CSF (93%). A several years’ follow-up of control patients, even positive ones for aSyn-SAA, did not show any signs attributable to synucleinopathies such as parkinsonism or cognitive decline except the RBD-NT1 patient with a young-onset parkinsonism.

**Table 1:** Clinical and demographic characteristics of recruited patients.

		N. pt	Sex	Age	RBD onset	RBD duration	Hyposmia	MDS-UPDRSIII	Ab NPS	Ab DatScan
			(m:f)	(years)	(years)	(years)	%		%	%
iRBD		41	29:12	70 ± 1	60 ± 2***	10 ± 1	49****	14 ± 4***	45 (13)	26 (35)
Controls	Iatrogenic and NT1-RBD	23	18:05	65 ± 3	49 ± 4	17 ± 5	0	3 ± 2	ND	10 (30)
	SD	6	04:02	63 ± 11	0	0	0	2 ± 1	0 (3)	0 (2)
	PN	11	07:04	70 ± 1	0	0	0	1 ± 0,5	ND	ND
Tot		40	29:11	66 ± 2	37 ± 5	15 ± 4	0	3 ± 2	0 (3)	9 (32)

*PN* peripheral neuropathy, *Ab* abnormal, *ND* not done; the number in brackets represents the number of patients in whom the test was performed.

***p < 0.01 (Student’s *t*-test).

****p < 0.001 (χ2).

### IF staining of pathological p-syn in skin nerves

**Fig. 1:**
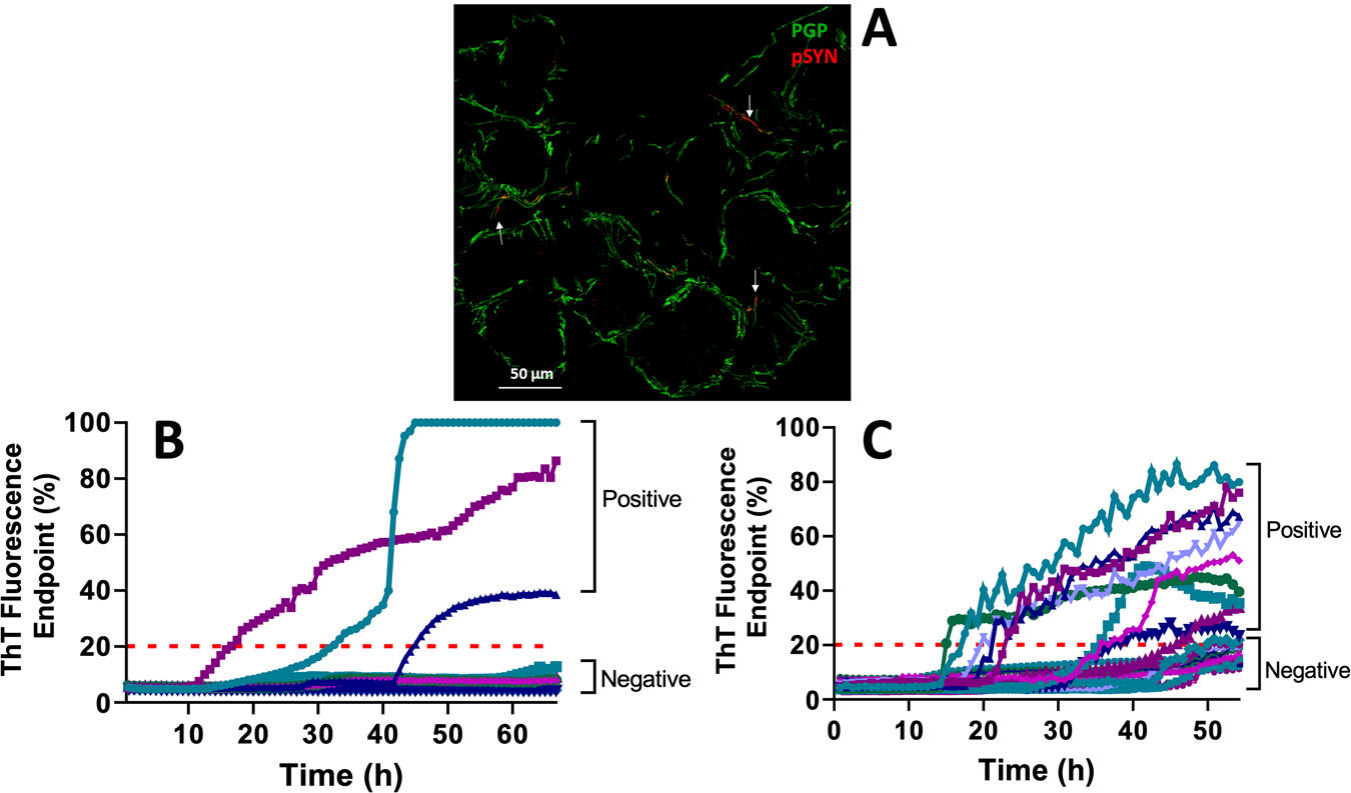
IF and aSyn-SAA findings. **A** *Phosphorylated α-synuclein (p-syn) deposits in autonomic skin nerves in a patient with iRBD*. Confocal microscope (400X) study of p-syn aggregates in skin nerves of a iRBD patient. Pathological p-syn deposits were found in autonomic nerves of a sweat gland. These fibers are identified by PGP 9.5 (in green) whereas p-syn aggregates were depicted by staining the phosphorylation at Ser 129 (in red). This merged image showed that abnormal p-syn deposits correspond to PGP 9.5 staining as neuritic inclusions (arrows). **B** *Pathological aSyn-SAA spectra of CSF from patients with RBD*. The pathological α-syn seeding activity of CSF from representative iRBD patients and RBD-NT1 patients or controls was detected by RT-QuIC assay. The positive (iRBD) and negative (RBD-NT1 patients) results are defined by the threshold (red dashed line) that is determined based on the average plus 4 standard deviations of non-iRBD controls. The ThT fluorescence response started to increase at approximately 20 h in samples from the iRBD patient, but not from the RBD-NT1 patients or controls. **C** *Skin pathological α-syn seeding activity tested by aSyn-SAA*. Skin αSynP fluorescence enhancement kinetics at each time point (0–55 h) from representative iRBD and control patients. The ThT fluorescence response was similar to that found for CSF and started to increase at approximately 15–20 h in samples from the iRBD patients.

P-syn positivity (Fig. 1) was disclosed in 32 patients with iRBD, and it was not found in any control patients with high diagnostic accuracy in disclosing a synucleinopathy (78% of sensitivity and 100% of specificity; Tables 2 and 3). Accordingly, iRBD patients and controls presented a different incidence of positive vs negative cases (*p* < 0.0001). Logistic regression model showed no influence of age, sex, and disease duration on the results.

**Table 2:** Diagnostic accuracy of IF and aSyn-SAA.

	IF skin	Skin aSyn-SAA	CSF aSyn-SAA
SENS	78%	59%	67%
SPEC	100%	82%	72%
PPV	100%	76%	72%
NPV	82%	67%	67%
ACC	89%	70%	67%

*SENS* sensitivity, *SPEC* specificity, *PPV* positive predictive value, *NPV* negative predictive value, *ACC* accuracy.

**Table 3:** IF and aSyn-SAA results in all recruited patients.

Cases	Diagnosis	Skin IF	CSF aSyn-SAA	Skin aSyn-SAA	Cases	Diagnosis	Skin IF	CSF aSyn-SAA	Skin aSyn-SAA
1	iRBD	POS	POS	NA	1	RBD-NT1	NEG	NEG	NA
2	iRBD	POS	NEG	NA	2	RBD-NT1	NEG	NEG	NA
3	iRBD	POS	NEG	NA	3	RBD-NT1	NEG	NEG	NA
4	iRBD	POS	NEG	NA	4	RBD-NT1	NEG	NEG	NA
5	iRBD	POS	NEG	NA	5	RBD-NT1	NEG	POS	NA
6	iRBD	POS	POS	NA	6	RBD-NT1	NEG	POS	NA
7	iRBD	POS	POS	NA	7	RBD-NT1	NEG	NEG	NA
8	iRBD	POS	NEG	NA	8	RBD-NT1	NEG	NEG	NA
9	iRBD	POS	NEG	NA	9	RBD-NT1	NEG	NEG	NA
10	iRBD	POS	NEG	NEG	10	RBD-NT1	NEG	NEG	NA
11	iRBD	POS	POS	POS	11	RBD-NT1	NEG	POS	NA
12	iRBD	POS	POS	NA	12	RBD-NT1	NEG	NEG	NA
13	iRBD	POS	POS	NA	13	RBD-NT1	NEG	NEG	NA
14	iRBD	POS	POS	NA	14	RBD-NT1	NEG	NEG	NA
15	iRBD	POS	NEG	NA	15	RBD-NT1	NEG	POS	NA
16	iRBD	POS	POS	NA	16	RBD-NT1	NEG	NEG	NA
17	iRBD	NEG	POS	NA	17	RBD-NT1	NEG	POS	NA
18	iRBD	POS	POS	NA	18	RBD-NT1	NEG	NEG	NA
19	iRBD	POS	POS	NA	19	RBD-NT1	NEG	POS	NEG
20	iRBD	POS	POS	NA	20	RBD-NT1	NEG	NEG	NEG
21	iRBD	NEG	POS	NA	21	RBD-NT1	NEG	NEG	NEG
22	iRBD	POS	POS	NEG	22	iatrog RBD	NEG	POS	NEG
23	iRBD	NEG	POS	POS	23	iatrog RBD	NEG	NEG	NEG
24	iRBD	POS	POS	POS	24	PN	NEG	NEG	NEG
25	iRBD	POS	POS	POS	25	PN	NEG	NEG	POS
26	iRBD	NEG	POS	POS	26	PN	NEG	POS	NEG
27	iRBD	POS	POS	POS	27	PN	NEG	NEG	NEG
28	iRBD	NEG	NEG	POS	28	PN	NEG	NEG	NEG
29	iRBD	NEG	POS	POS	29	PN	NEG	NEG	NEG
30	iRBD	POS	POS	POS	30	PN	NEG	NEG	POS
31	iRBD	POS	POS	POS	31	PN	NEG	NEG	NEG
32	iRBD	NEG	POS	POS	32	PN	NEG	NEG	NEG
33	iRBD	NEG	NEG	NEG	33	PN	NEG	POS	NEG
34	iRBD	POS	NEG	NEG	34	PN	NEG	NA	POS
35	iRBD	POS	POS	NEG	35	OSAS	NEG	NA	NEG
36	iRBD	POS	POS	NEG	36	OSAS	NEG	POS	POS
37	iRBD	POS	NEG	NEG	37	OSAS	NEG	NEG	NEG
38	iRBD	POS	NA	POS	38	OSAS	NEG	NA	NEG
39	iRBD	POS	NA	NEG	39	OSAS	NEG	NEG	NEG
40	iRBD	NEG	NEG	POS	40	OSAS	NEG	NA	NEG
41	iRBD	POS	POS	NEG					

RBD-NT1 patients 6, 7, and 9 showed abnormal nigro-striatal DatScan, in addition, RBD-NT1 patient 6 also showed young-onset parkinsonism.

*POS* positive, *NEG* negative, *iRBD* idiopathic REM sleep behavior disorder, *RBD-NT1* RBD associated with type 1 Narcolepsy, *Iatrog RBD* RBD associated with iatrogenic causes, *OSAS* obstructive apnea during sleep, *NA* not available.

### aSyn-SAA evaluation of pathological α-syn seeding activity in skin and CSF samples

A positive α-syn seeding activity (Fig. 1) was found in 13 iRBD patients and in 4 controls (3 PN and 1 OSAS) in skin samples and 26 iRBD but also in 10 controls (7 iatrogenic and NT1-RBD, 2 PN and 1 OSAS) in CSF yielding a lower diagnostic accuracy than IF (Tables 2 and 3). However, the skin showed higher aSyn-SAA diagnostic accuracy than CSF (Tables 2 and 3). As for IF iRBD patients and controls showed a different incidence of positive vs negative cases (*p* < 0.01). Regression models showed no influence of age, sex, and disease duration on the results.

### IF vs aSyn-SAA to detect pathological α-syn

Considering all examined cases p-syn staining in skin nerves disclosed by IF showed a weak agreement with CSF aSyn-SAA (Kappa = 0.3; *p* < 0.01) whereas no agreement was found between IF and skin aSyn-SAA (Kappa = 0.1; *p* = 0.4). The highest agreement was found between skin and CSF aSyn-SAA (Kappa = 0.4; *p* < 0.05).

### Side effects of used procedures

#### Skin biopsy

Minor bleeding occurs in 5 out of 81 patients investigated (6%) which was resolved with a containment dressing without requiring suture.

#### Lumbar puncture

Orthostatic headache has been reported in 13 patients out of 75 patients investigated (17%) who significantly changed the habits of the patients (as they were unable to work) and required the prolonged use of a drug therapy (paracetamol or NSAID) for several days.

## Discussion

The main results of our study in disclosing an underlying pathological form of α-syn in iRBD are that: (1) skin IF presented a higher diagnostic accuracy than aSyn-SAA because of a lower sensitivity and specificity; (2) however, IF presented a modest level of agreement with CSF aSyn-SAA supporting their convertibility to disclose a pathological form of α-syn.

iRBD is the most reliable clinical marker of prodromal synucleinopathies since the majority of patients with iRBD are diagnosed with any synucleinopathy within 20 years of onset of iRBD as supported by disclosing a pathological form of α-syn in skin samples of the majority of patients investigated^3,8^. By contrast, NT1 is believed to be caused by an autoimmune mechanism^9,10^. RBD in NT1 patients has been associated with nocturnal-motor activity/behavior recurred with an almost stereotypic pattern, through all the night in REM sleep likely reflecting the typical instability of sleep with dissociates states resulting from hypocretin deficiency^8^. NT1-RBD does not show in vivo pathological accumulation of pathological α-syn^11^ representing the ideal control of diagnostic tools aimed to disclose pathological form of α-syn in iRBD. PN who did present clinical signs and symptoms of neurodegenerative disorders were also included as controls to ascertain a specificity of findings disclosed in iRBD.

The correct in vivo diagnosis of synucleinopathies is a major challenge with prognostic and therapeutic implications, particularly in the early disease phase when the start of a specific treatment will be particularly important to prevent the effect of the underlying neurodegenerative pathological diffusion. Thus, a reliable biomarker for synucleinopathies in the early disease phase will be particularly helpful to develop a disease-modifying therapy.

IF and aSyn-SAA are two diagnostic tools recently developed to disclose in vivo pathological α-syn. However, they explore different properties of pathological α-syn since IF may detect the morphology of pathological aggregates in skin nerves, whereas aSyn-SAA can determine their seeding activity, a prion-like biological trait of misfolded proteins^7^. Thus, the diagnostic accuracy of each test in disclosing the pathological form of α-syn may differ in patients with iRBD and a direct comparison it should be performed to identify the diagnostic accuracy of these tests.

Our data showed that the IF presented higher diagnostic accuracy than aSyn-SAA mainly because absent positivity in the controls. By contrast, aSyn-SAA, mainly skin, exhibited an acceptable sensitivity in disclosing abnormal α-syn in the iRBD while it showed a lower specificity because of some of positive results in the control group. Accordingly, skin aSyn-SAA showed higher diagnostic accuracy than CSF aSyn-SAA. aSyn-SAA in the skin showed a similar diagnostic accuracy than our previous study made in full-blown α-synucleinopathies^7^ whereas it was lower in CSF did not confirming the previous promising diagnostic accuracy in CSF recently reported even in iRBD^12^. The difference could be explained by experimental procedures mainly related to the different recombinant α-synuclein used. We used a commercially available recombinant α-synuclein whereas a home-made protein was used in other studies^12^. The variable result of aSyn-SAA based on different recombinant α-synuclein sources should be addressed in the future with the aim of optimizing the experimental procedures for aSyn-SAA. The use of skin samples by aSyn-SAA is then a helpful option for the future due to a less invasive procedure than CSF, obtained by a lumbar puncture, which has fewer side effects as also confirmed in this study.

The reason of pathological α-synuclein positivity in controls remains to be clarified but it could be due to an incidental synucleinopathy, although this possibility was not found in previous studies in NT1^13,14^. We carefully re-evaluated control patients found to be positive at the aSyn-SAA, particularly with RBD-NT1, but no clinical signs attributable to a synucleinopathy could be found expect the patient with a young-onset parkinsonism who probably does not present a classic synucleinopathy considering the lack of LDopa response during a 15 years of neurological follow-up. In addition, the slight abnormal nigro-striatal DatScan found in RBD-NT1 was not associated with the α-synuclein seeding positivity as the abnormality was more frequent in negative aSyn-SAA patients.

The current study and a previous study we have made in full-flown variants of synucleinopathies^7^ supported the use of IF and aSyn-SAA as specific tools to disclose in vivo pathological α-synuclein as biomarker for these disorders.

Finally, skin IF may also be appropriate for the early identification of a specific variant of synucleinopathy in iRBD patients as previous data showed the possibility to differentiate Parkinson disease or dementia with Lewy bodies from Multiple System Atrophy based on a prevalent deposition of misfolded α-syn in autonomic or somatic skin nerves^15,16^ or skin Schwann cells^17^.

This study presents the following limitations: (1) no data regarding the progression and phenoconversion of our iRBD patients into a specific form of synucleinopathy. However, the predictive value of misfolded synuclein in iRBD as a biomarker of phenoconversion has been established by previous papers for both IF^18^ and aSyn-SAA^19^; (2) the lack of autopsy-confirmed diagnosis although the clear-cut differences we have found by IF among the investigated patients may support that the selection was made correctly; (3) the suboptimal aSyn-SAA we obtained in CSF can prevent a reliable comparison between CSF and skin assays. Further studies using fully optimized aSyn-SAA in CSF and skin are needed to make a comparison between the diagnostic accuracy of different samples in disclosing pathological α-synuclein by SAA.

## Methods

We prospectively screened 41 patients with iRBD, based on current diagnostic criteria^8^ and 23 matched patients who came to our observation for a RBD as an impacting clinical symptom and finally received the diagnosis of type 1 Narcolepsy (NT1, 21 patients) or an iatrogenic RBD (2 patients) according to current criteria^8^. A 77-years-old RBD-NT1 patient presented a young-onset (32 years) parkinsonism not responding to Ldopa treatment. In addition, 11 patients with peripheral neuropathies suspected to be autoimmune (PN) and 6 patients with obstructive apnea during sleep (OSAS) who underwent to puncture lumbar were also recruited as controls without a supposed neurodegenerative disorder. Patients with RBD underwent video– polysomnography (PSG) recording which documented REM sleep without atonia and at least one episode of RBD. Patients underwent extensive examinations, with complete clinical and neurological examination, neuropsychological investigations by means of a brief mental deterioration battery (BMDB), a neuropsychological test standardized in the Italian population expressing a global cognitive impairment when the final score is negative (<0)^20^, brain MRI, nigrostriatal dopamine transporter ligand [123I]ioflupane-DaTscan (Table 1). Hyposmia was reported by patients during the neurological examination (Table 1).

Patients with alcohol-use disorder, taking antidepressants, or beta-blockers or with signs of motor or cognitive dysfunctions were excluded.

The study has been approved by the local Human Ethics Committee (Comitato Etico Indipendente-AVEC, Azienda USL Bologna and Imola, n. 13004/Sper/AUSLBO) and followed the Helsinki Declaration regarding international clinical research involving human beings. All subjects gave their written informed consent to the study.

### IF staining of pathological p-syn in skin nerves

Three mm punch biopsies were taken from proximal and distal hairy skin sites. The proximal site included the cervical C7 paravertebral area bilaterally whereas distal sites were located in the distal leg bilaterally (10 cm above the lateral malleolus). As previously reported, we took in each skin site a second biopsy approximately 3 centimetres away from the first sample to increase the rate of p-syn positivity^4,21^. IF analysis was made blinded to the clinical diagnosis.

According to previously published procedures^21,22^ skin samples were immediately fixed in cold Zamboni’s fixative and kept at 4 ^o^C overnight.

Ten μm sections were obtained using a cryostat (CM 1950; Leica, Deerfield, IL). They were double-immunostained overnight with a panel of primary antibodies including rabbit monoclonal phosphorylated α-synuclein at Ser 129 (p–syn; 1:500, abcam, Cambridge, UK, cat. no. ab-51253) and mouse pan-neuronal marker protein gene product 9.5 (1:750; Abcam, Cambridge, UK, cat. no. ab72911). Sections were then washed and secondary antibodies were added for an incubation of 1 h. As for secondary antibodies, an anti-mouse Alexa Fluor(R) 488 (1:400; Jackson ImmunoResearch, West Grove, PA, USA, cat. num. 715-545-150) and rabbit cyanine dye fluorophores 3.18 (1:200, Jackson ImmunoResearch, West Grove, PA, USA; cat. num. 711-165-152) were used. The microscope analysis and criteria followed to define a p-syn positivity were previously described^7,21–23^. Shortly, the correspondence between rabbit p-syn and mouse PGP staining helped verify the intraneuronal deposits, excluding possible non-specific staining arising from the background. The analysis was made in a blinded fashion to the clinical diagnosis by two authors with expertise in immunofluorescent analysis (DV and IA). The intra-laboratory analysis revealed an excellent reproducibility, with a 100% concordance of classification in all patients (K = 1), in agreement with recently reported data^24^. P-syn staining was rated as positive when a single skin nerve fiber showed a positive staining at high magnification (x40) in the two close skin samples. The co-localization of p-syn with PGP we also analysed by acquiring digital images using a laser-scanning confocal microscope (Nikon confocal microscopy, Eclipse Ti A1, Japan). Each image was collected in successive frames of 1–2 μm increments on a Z-stack plan at the appropriate wavelengths for secondary antibodies with a x20 or x40 plan apochromat objective and subsequently projected to obtain a double-stained digital image by a computerized system (LCS lite, Leica Microsystems, Heidelberg, Germany).

### aSyn-SAA analysis of seeding activity of pathological α-synuclein in skin and CSF

#### Skin

The skin punch biopsy samples (~30 mg each in weight, ~3 × 3 mm each in size) included epidermis and dermis layers. The two frozen skin samples taken from C7 site 2–3 cm apart from the pair of skin samples used for IF^7^ were additionally analysed in 22 iRBD and 22 control patients (5 iatrogenic and NT1-RBD, 11 PN and 6 OSAS). The two frozen skin samples were prepared for aSyn-SAA analysis as described previously^7^. Each single skin sample was analysed individually. The skin tissues washed for three times in 1x Tris-buffered saline (TBS) were separated into small pieces in dishes. The skin homogenates at 10% (w/v) were prepared in skin lysis buffer containing 2 mM CaCl2 and 0.25% (w/v) collagenase A (Roche) in TBS and incubated at 37 °C for 4 h with shaking, followed by homogenization in a Mini-Beadbeater (BioSpec, Laboratory Supply Network, Inc., Atkinson, NH, USA) for 1 min. After sonication, the samples were centrifuged at 500 g for 3 min to collect the supernatant fraction (S1). aSyn-SAA reaction mix was composed of 40 mM phosphate buffer (pH 8.0), 170 mM NaCl, 0.1 mg/mL recombinant human wild-type α-syn purchased commercially (rPeptide, Watkinsville, GA, USA), 10 µM Thioflavin T (ThT), and 0.00125% SDS. Aliquots of the 98 µL or 85 µL reaction mix/each were loaded into each well of a black 96-well plate with a clear bottom (Nunc) preloaded with ~5 glass beads (diameter = 1 mm) and seeded with 2 µL of skin homogenate S1. The skin homogenates were spun at 2000 g for 2 min at 4 °C prior to making serial dilutions. The plate sealed with a plate sealer film (Nalgene Nunc International) was incubated at 42 °C in a BMG FLUOstar Omega plate reader with cycles of 1 min shaking (400 rpm double orbital) and 1 min rest for the indicated incubation time. The measurements of ThT fluorescence (450 + /−10 nm excitation and 480 + /−10 nm emission; bottom read) were conducted every 45 min. Four replicate reactions were seeded with the same dilution for each individual sample. The average fluorescence value of each sample was calculated using fluorescence values from all four replicate wells regardless of whether these values crossed the threshold described below. The maximal fluorescence response (260.000 rfu) of the plate readers was plotted versus different time points of measurements. Skin aSyn-SAA was considered positive in case of reaction of at least one of two analysed samples. The aSyn-SAA fluorescence signal reported for the analysis from each skin site was the biggest value of the two samples analysed.

#### CSF

It was analysed in 39 iRBD and 36 control patients (23 iatrogenic and NT1-RBD, 10 PN and 3 OSAS). A total of ~60–100 µL aliquot of CSF for each patient was kept at −80 °C in a freezer for aSyn-SAA. In brief, aSyn-SAA reaction mix was composed of 40 mM phosphate buffer (pH 8.0), 170 mM NaCl, 0.1 mg/ml recombinant human wild-type α-syn purchased commercially (rPeptide, Watkinsville, GA, USA), 10 µM Thioflavin T (ThT), and 0.00125% SDS. Aliquots of the 98 µL or 85 µL reaction mix/each were loaded into each well of a black 96-well plate with a clear bottom (Nunc) preloaded with ~5 glass beads (diameter = 1 mm) and seeded with 2 µL of 15 µL of CSF. The CSF samples were spun at 2.000 g for 2 min at 4 °C prior to making serial dilutions. The plate sealed with a plate sealer film (Nalgene Nunc International) was incubated at 42 °C in a BMG FLUOstar Omega plate reader with cycles of 1 min shaking (400 rpm double orbital) and 1 min rest for the indicated incubation time. The measurements of ThT fluorescence (450 + /−10 nm excitation and 480 + /−10 nm emission; bottom read) were conducted every 45 min. Four replicate reactions were seeded with the same dilution for each individual sample. The average fluorescence value of each sample was calculated using fluorescence values from all four replicate wells regardless of whether these values crossed the threshold described below. The maximal fluorescence response (260.000 rfu) of the plate readers was plotted versus different time points of measurements. aSyn-SAA reaction mix was composed of 40 mM phosphate buffer (pH 8.0), 170 mM NaCl, 0.1 mg/mL recombinant human wild-type α-syn purchased commercially (rPeptide, Watkinsville, GA, USA), 10 µM Thioflavin T (ThT), and 0.00125% SDS. Aliquots of the 98 µL or 85 µL reaction mix/each were loaded into each well of a black 96-well plate with a clear bottom (Nunc) preloaded with ~5 glass beads (diameter = 1 mm) and seeded with 2 µL of CSF.

The skin and CSF cut-off point for defining the positive seeding activity was the same already described and based on the mean ThT value of control samples at 60 h, plus 3 standard deviations^7^. The analysis was made blinded to the clinical diagnosis.

### Statistical analysis

Statistical analyses were performed using SPSS 25.0. iRBD and control patients demographic data were compared by Student’s *t*-test as the data were normally distributed. aSyn-SAA data were analyzed with GraphPad Prism version 8.4.3 (GraphPad Software). For comparison of categorical variables, we used χ2 test (or Fisher’s exact test, when appropriate) and presented the results as absolute and relative frequency (%). To adjust the results for age, gender, and disease duration, we further used logistic or multivariate linear regression depending on the data. Agreement was tested using Cohen’s k statistics. For all analyses, significance was assumed for *p* < 0.05; Bonferroni-corrected for multiple comparisons.

### Reporting summary

Further information on research design is available in the Nature Research Reporting Summary linked to this article.

## Supplementary information

Reporting Summary

## Data Availability

The datasets generated and analysed during the current study are available from the corresponding authors upon request.

## References

[1] Dauvilliers Y (2018). REM sleep behaviour disorder. Nat. Rev. Dis. Prim..

[2] Antelmi E, Donadio V, Incensi A, Plazzi G, Liguori R (2017). Skin nerve phosphorylated α-synuclein deposits in idiopathic REM sleep behavior disorder. Neurology.

[3] Doppler K (2017). Dermal phospho-alpha-synuclein deposits confirm REM sleep behaviour disorder as prodromal Parkinson’s disease. Acta Neuropathol..

[4] Donadio V (2019). Skin nerve α-synuclein deposits in Parkinson’s disease and other synucleinopathies: a review. Clin. Auton. Res.

[5] Groveman BR (2018). Rapid and ultra-sensitive quantitation of disease-associated α-synuclein seeds in brain and cerebrospinal fluid by αSyn RT-QuIC. Acta Neuropathol. Commun..

[6] Wang Z (2020). Skin α-Synuclein aggregation Seeding Activity as a Novel Biomarker for Parkinson’s disease. JAMA Neurol.

[7] Donadio V (2021). In vivo diagnosis of synucleinopathies: a comparative study of skin biopsy and RT-QuIC. Neurology.

[8] American Academy of Sleep Medicine. International classification of sleep disorders. Diagnostic and coding manual. 3rd ed. Westchester, IL: American Academy of Sleep Medicine; (2014).

[9] Partinen M (2014). Narcolepsy as an autoimmune disease: the role of H1N1 infection and vaccination. Lancet Neurol..

[10] Bassetti CLA (2019). Narcolepsy - clinical spectrum, aetiopathophysiology, diagnosis and treatment. Nat. Rev. Neurol..

[11] Antelmi E (2019). Biomarkers for REM sleep behavior disorder in idiopathic and narcoleptic patients. Ann. Clin. Transl. Neurol..

[12] Rossi M (2020). Ultrasensitive RT-QuIC assay with high sensitivity and specificity for Lewy body-associated synucleinopathies. Acta Neuropathol..

[13] Jennum PJ (2017). Cerebrospinal fluid biomarkers of neurodegeneration are decreased or normal in narcolepsy. Sleep.

[14] Baiardi S (2020). Cerebrospinal fluid biomarkers of neurodegeneration in narcolepsy type 1. Sleep.

[15] Donadio V (2018). Skin α-synuclein deposits differ in clinical variants of synucleinopathy: an in vivo study. Sci. Rep..

[16] Donadio, V. et al. Skin biopsy may help to distinguishing MSA-P from Parkinson’s disease with orthostatic hypotension. *Mov Disord.*10.1002/mds.28126 (2020). 10.1002/mds.28126 32557839

[17] Donadio, V. et al. Phosphorylated α-synuclein in skin Schwann cells: a new biomarker for multiple system atrophy. *Brain* awac 124. 10.1093/brain/awac124 (2022). 10.1093/brain/awac124 35552610

[18] Doppler K (2021). Consistent skin α-synuclein positivity in REM sleep behavior disorder—A two center two-to-four-year follow-up study. Parkinsonism Relat. Disord..

[19] Iranzo A (2021). Detection of α-synuclein in CSF by RT-QuIC in patients with isolated rapid-eye-movement sleep behaviour disorder: a longitudinal observational study. Lancet Neurol..

[20] Gallassi R (1986). Neuropsychological assessment of mental deterioration: purpose of a brief battery and a probabilistic definition of ‘normality’ and ‘non-normality’. Acta Psychiatr. Scan.

[21] Donadio V (2018). Skin nerve phosphorylated α-synuclein deposits in Parkinson’s disease with orthostatic hypotension. J. Neuropathol. Exp. Neurol..

[22] Donadio V (2018). Skin nerve phosphorylated α-synuclein deposits in Parkinson’s disease with orthostatic hypotension. J. Neuropathol. Exp. Neurol..

[23] Donadio V (2013). Skin sympathetic fiber α-synuclein deposits: a potential biomarker for pure autonomic failure. Neurology.

[24] Donadio V (2019). Abnormal α-synuclein deposits in skin nerves: intra- and inter-laboratory reproducibility. Eur. J. Neurol..

